# Identification of a HIV Gp41-Specific Human Monoclonal Antibody With Potent Antibody-Dependent Cellular Cytotoxicity

**DOI:** 10.3389/fimmu.2018.02613

**Published:** 2018-11-16

**Authors:** Zheng Yang, Xi Liu, Zehua Sun, Jingjing Li, Weiguo Tan, Weiye Yu, Meiyun Zhang

**Affiliations:** ^1^Department of Tuberculosis Prevention, Shenzhen Center for Chronic Disease Control, Shenzhen, China; ^2^AIDS Institute, Department of Microbiology, Li Ka Shing Faculty of Medicine, The University of Hong Kong, Hong Kong, Hong Kong; ^3^Department of Infectious Diseases, The Fifth Affiliated Hospital, Sun Yat-sen University, Zhuhai, China; ^4^National Jewish Health, Denver, CO, United States

**Keywords:** antibody, HIV, ADCC, epitope, gp41, MPER

## Abstract

Antibody-Dependent Cellular Cytotoxicity (ADCC) is a major mechanism of protection against viral infections *in vivo*. Identification of HIV-1-specific monoclonal antibodies (mAbs) with potent ADCC activity may help develop an effective HIV-1 vaccine. In present study, we isolated such human mAb, designated E10, from an HIV-1-infected patient sample by single B cell sorting and single cell PCR. E10 bound to gp140 trimer and linear peptides derived from gp41 membrane proximal external region (MPER). E10 epitope (QEKNEQELLEL) overlapped with mAb 2F5 epitope. However, E10 differentiated from 2F5 in neutralization breadth and potency, as well as ADCC activity. E10 showed low neutralization activity and narrow spectrum of neutralization compared to 2F5, but it mediated higher ADCC activity than 2F5 at low antibody concentration. Fine mapping of E10 epitope may potentiate MPER-based subunit vaccine development.

## Introduction

Antibody-Dependent Cellular Cytotoxicity (ADCC) has been repeatedly reported to be able to provide protection against HIV/AIDS. Antibodies that mediate ADCC activity can decrease viral load (1, 2), lower infection risk (3, 4), reduce transmission rate (5), slower disease progression (6–8), reduce HIV-infected infants' mortality (9) and clear latent HIV reservoir after reactivation (10, 11). RV144 HIV vaccine phase III clinical trial demonstrated that high level of ADCC antibody correlated with decreased infection risk (3, 12). Identifying HIV-1 mAbs with potent ADCC activity and developing HIV-1 vaccine immunogens that can elicit such ADCC antibodies has become a new research area in HIV/AIDS vaccine field (13–17).

According to our and others' research, broadly neutralizing HIV-1 mAbs may not confer potent ADCC activity. Instead, non-neutralizing mAbs may mediate high ADCC activity, suggesting that neutralizing epitopes may be different from ADCC epitopes. More than a dozen of HIV-1 broadly neutralizing mAbs have been characterized for epitopes identified (18).Antibody b12 was isolated from a long term none progressor derived combinatorial antibody phage display library (19, 20). It neutralized about 75% of clade B primary viruses and 41% of 190 isolates, representing major HIV-1 clades (21). This antibody recognizes a highly conserved epitope overlapping the CD4-binding region of gp120, which is responsible for its broad recognition of different HIV-1 isolates (19). IgG1 b12 was found to be able to mediate ADCC activity in macaques model (22). Passive immunization of mAb b12 in rhesus macaques decreased viral load through ADCC as its protective activity is diminished in the absence of FcR binding capacity (1). 2F5 is a broadly neutralizing anti-HIV-1 IgG isolated from sera of naturally asymptomatic infected individuals (23). It recognizes the well-conserved hexapeptide ELDKWA, the central region of the larger NEQELLELDKWASLWN sequence of the MPER (24). 2F5 mediated ADCC against both X4 and R5 tropic envelope at low concentration (25). MAb VRC01 was isolated by single B cell sorting and PCR, it recognize the CD4 binding site of gp120. The neutralization potency and breath of VRC01are much better than that of 2F5 and b12 (21), however its ADCC activity is not significant (26, 27). The non-neutralizing ADCC antibody A32 targeted the CD4 inducible conformational epitopes on C1of gp120. It mediated ADCC activity that was four- to six-fold higher than that of MAbs b12 and VRC01 (27). A32-like antibodies was believed to be responsible for the majority of ADCC activity present in HIV+ sera and linked to decreased HIV-1 transmission in the RV144 trial (3, 28). A32-like epitopes were regarded as major ADCC epitopes on HIV envelop protein (29).

Single B cell sorting and antibody gene amplification platform have been a conventional tool for antibody identification (16, 30, 31, 32). High potency broad neutralizing antibodies like VRC01 (21), PGT121 (33), CH103 (34), and 10E8 (35) are all isolated by single B cell sorting technology. In present study, we applied this technology to identifying HIV-1 mAbs with high ADCC activity from memory B cells prepared from HIV infected individuals. The isolated mAbs were characterized for binding affinity, neutralization activity and ADCC activity, as well as epitope localization.

## Results

### Identification of HIV envelope specific antibody E10 by single B cell sorting and single cell PCR

**Figure 1:**
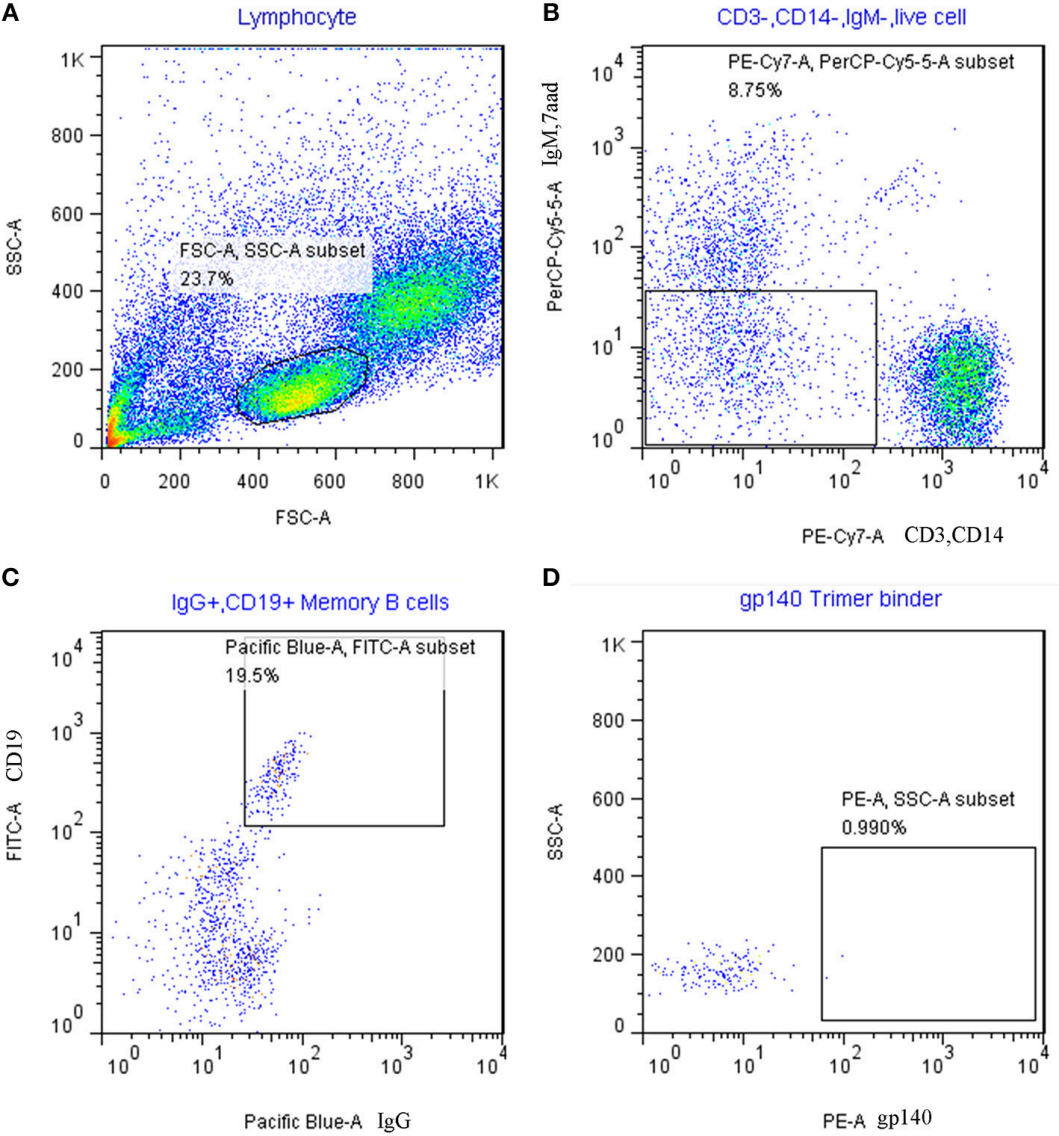
Single cell sorting of gp140 trimer specific memory B cell from PBMC of HIV infected patient. **(A)** gate on lymphocyte; **(B)** gate on IgM (PerCP-cy5.5) negative, 7AAD (PerCP-cy5.5) negative, CD3(PE-CY7) negative and CD14 (PE-CY7) negative population; the purpose is to remove IgM positive cells and T cells. **(C)** Gate on CD19 (FITC) positive and IgG (Pacific blue) positive population; the purpose is to gate the IgG positive B cells. **(D)** Gate on SF162gp140 trimer binder (PE), the purpose is to fish out antigen specific IgG positive B cells.

**Figure 2:**
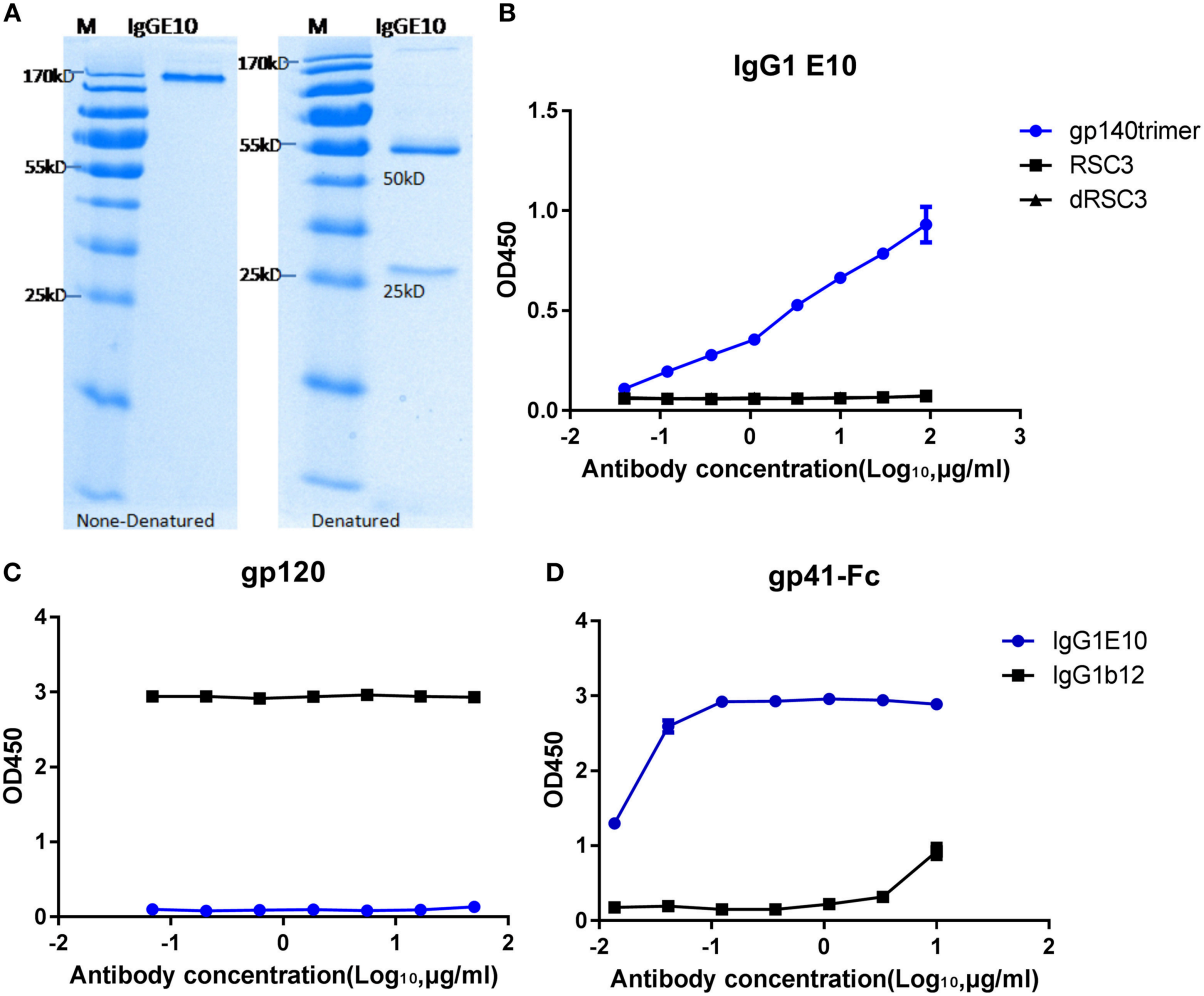
Affinity detection of Mab E10 against gp120 (bal) and gp41-Fc (bal) by ELISA. **(A)** IgG E10 size and purity detection by denatured and none-denatured SDS-PAGE; binding **(B)** of IgG E10 against SF162gp140 trimer, RSC3 and delta RSC3; **(C)** binding of IgG E10 against bal gp120; **(D)** binding of IgG E10 against gp41-Fc fusion protein.

Forty eight HIV envelope specific single memory B cells (CD19+, IgG+, SF162gp140trimer +, 7AAD-, IgM-, CD14-, and CD3-) were sorted from PBMC of HIV patient (Figure 1). After cell lysis, RNA reverse transcription and antibody fragment amplification, 10 pairs of antibody gene fragments were recovered and 8 pairs of antibody vectors were successfully constructed. The heavy chain and light chain containing vectors were co-transfected to 293F cells and then full length IgG1 were purified from the culture supernatant. The purified IgGs show correct size and high purity on both denatured and none-denature SDS-PAGE (Figure 2A). One of the purified IgG antibody (Mab E10) show high binding to SF162 gp140 trimer protein(Figure 2B) and balgp41-Fc protein (Figure 2D), but not to balgp120 (Figure 2C). It indicates that E10 is a gp41 specific antibody. The EC50 of its affinity to bal gp41-Fc is about 0.05 μg/ml.

### Mab E10 showed low neutralization activity compared to B12 and 2F5

**Figure 3:**
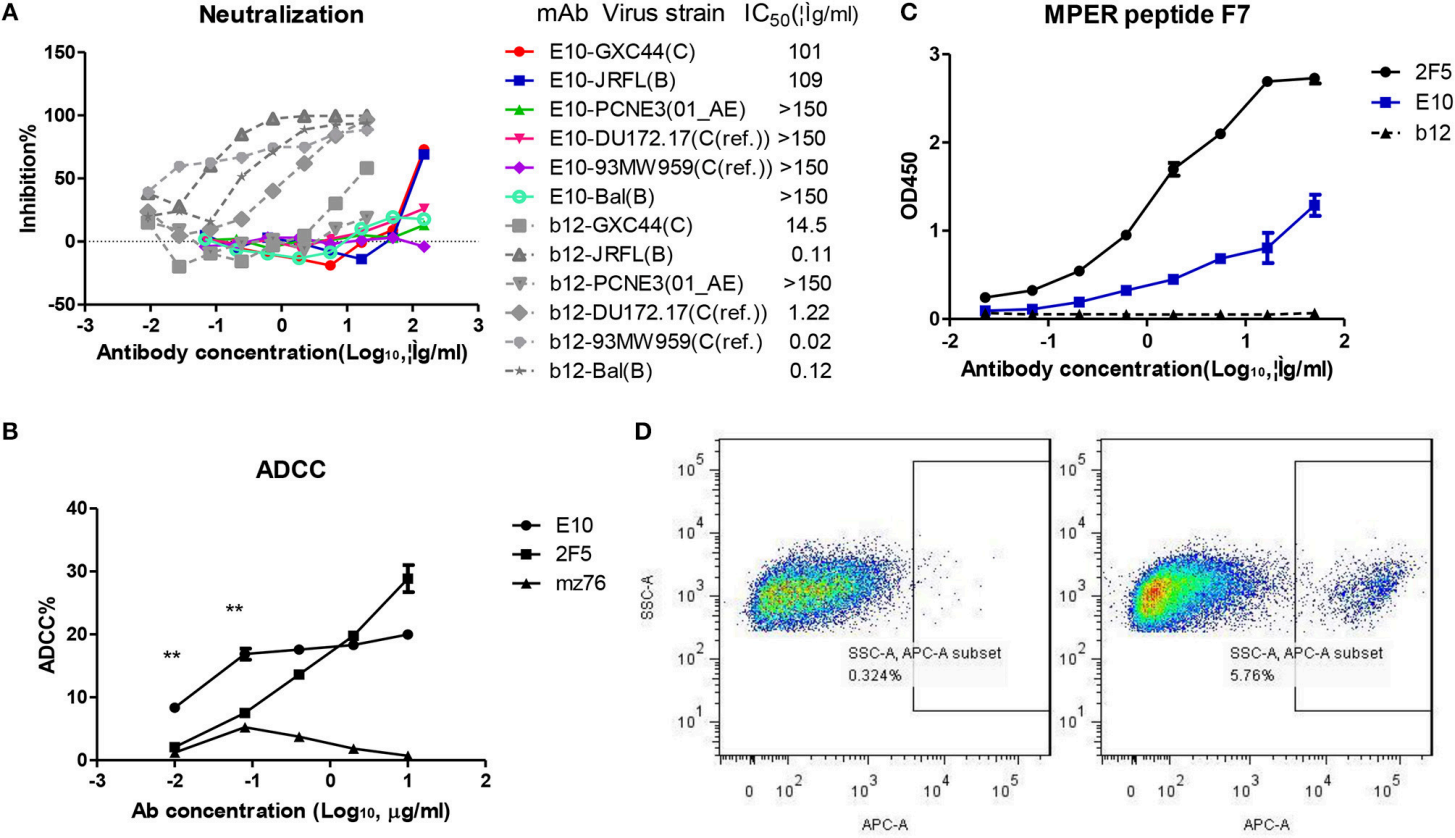
Neutralization and ADCC activity of IgG E10 and its binding to MPER peptide F7. **(A)** Infection inhibition percentage of IgG b12 and E10 against 6 pseudo type HIV strains at different concentrations were shown, calculated IC50 in μg/ml were labeled after the strain and clade names. **(B)** IgG1 E10, 2F5, and mz76 mediated cellular cytotoxicity against HIV envelope expressing TF228 at different concentrations (μg/ml) were shown by percentage. Experiments were repeated three times. **(C)** Affinity detection of IgG E10, 2F5, and b12 to MPER peptide fusion protein F7-Fc by ELISA; OD450 at different antibody concentration were shown. **(D)** FlowJo generated figure showing binding of E10 SCFV expression yeast clone with bal gp120-Fc and F7-Fc fusion proteins, APC positive population indicate positive binding. Statistical analysis, ^**^P ≤ 0.01.

Despite high affinity of E10 for gp41, it exhibited low neutralization potency compared to b12 and 2F5. We tested E10 for neutralization activity against GXC44(C), JRFL (B), PCNE3 (01_AE), Du172.17[C (ref.)], and 93MW959 (C (ref.)) by HIV-1 pseudo virus neutralization assay. Mab E10 can neutralize GXC44(C) and JRFL (B), but with high IC50s (101, 109 μg/ml, respectively) (Figure 3A). It did not neutralize the other four isolates.On the contrary, the control neutralizing antibody b12 can neutralize 5/6 isolates in this panel with IC50 <20 μg/ml (Figure 3A). Despite its high affinity against gp41, Mab E10's neutralization potency and breath is comparatively low. The epitopes of Mab E10 may not be neutralizing epitopes.

### Mab E10 showed high ADCC activity at low concentrations

With TF228 as target cell and pooled healthy donor PBMC as effector cell, IgG1 E10mediated ADCC activity was compared with IgG1 2F5 and IgG1 MZ76 (V5 loop specific, manuscript in preparation).Unlike the proverbial neutralizing and ADCC antibody IgG 2F5, Mab E10 mediated higher ADCC activity at low antibody concentrations (below 0.1 μg/ml). Interestingly, ADCC activity of E10 did not increase significantly when antibody concentration exceeded 0.1 μg/ml, suggesting steric hindrance to E10 binding (Figure 3B). The control antibody IgGMZ 76 showed low ADCC activity at all four doses tested (Figure 3B).The high ADCC activity of Mab E10 at low concentration may potentiate its usage in prevention and treatment of HIV-1 infection.

### Mab E10 bound to a linear epitope on gp41

The affinity ELISA results has confirmed that Mab E10 is gp41 specific. To localize E10 epitope, we constructed a yeast antigen library which displays HIV-1 envelope fragments. The yeast clones that specifically bind to Mab E10 were sorted and re-cultured. After yeast plasmid extraction and sequencing, we got a highly enriched sequence that encodes peptide F7 (amino acid: QQELLELDKWANLWNWFDISNWLW, 657–680, HXB2 numbering). To confirm the binding of E10 to F7 peptide, E10 were constructed to yeast vector PYD7 as single chain form. It can display on the surface of EBY100 recombinant yeast after galactose induction. F7 peptide were expressed as human Fc fusion protein and purified by protein G agarose. The binding of E10 toF7-Fc protein was confirmed by F7 ELISA (Figure 3C); the binding of E10 SCFV surface displayed yeast to F7-Fc was confirmed by FACS (Figure 3D).

**Figure 4:**
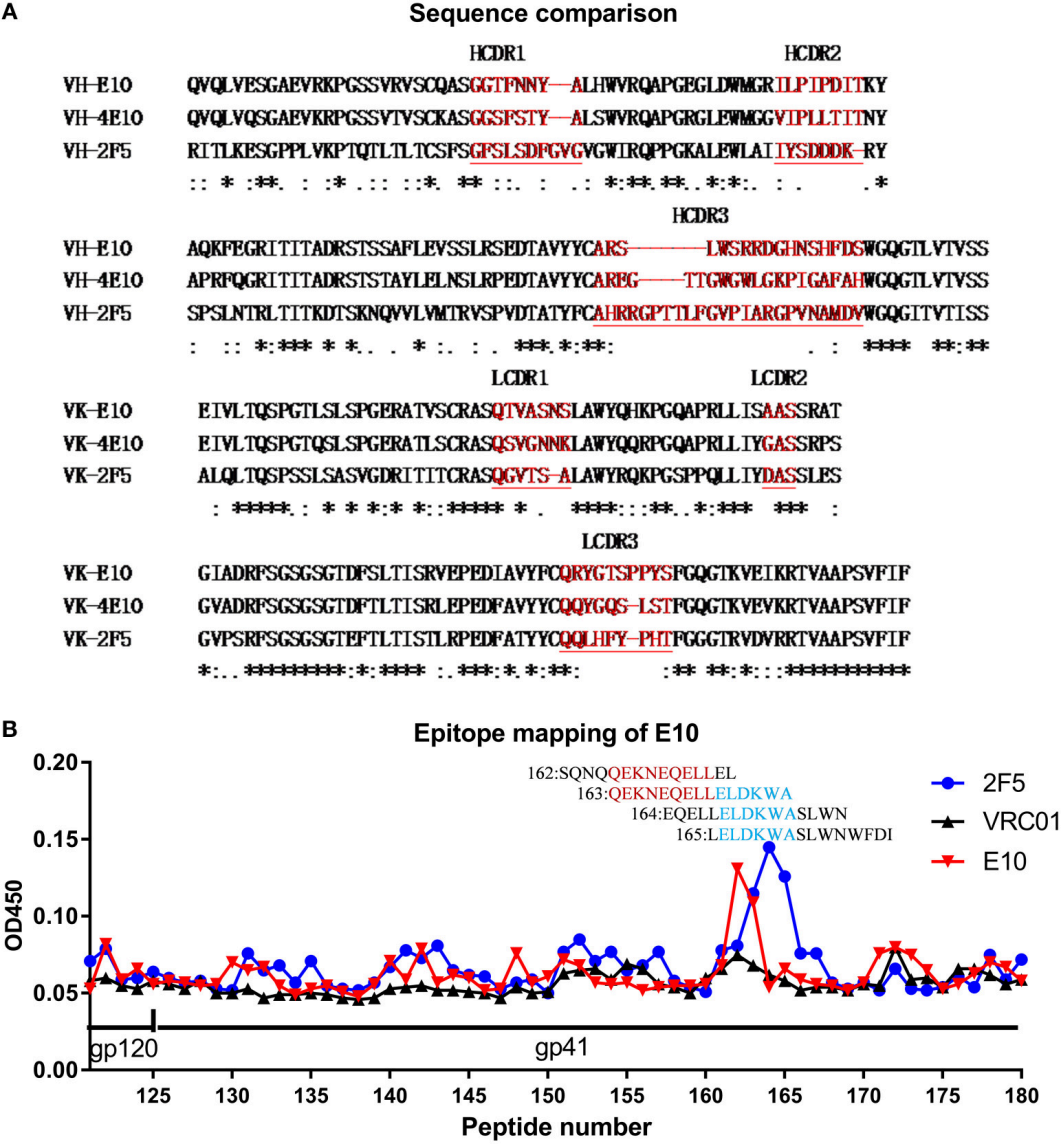
Epitope amino acid sequence comparison between mAb E10, 2F5 and 4E10 **(A)** and epitope mapping of mAb E10 by gp41 peptide microarray ELISA **(B)**. CDR sequences of antibodies in bright red **(A)**, peptides bind by antibodies were labeled with number and sequence. E10 core epitope in dark red and 2F5 core epitope in bright blue **(B)**.

F7 peptide contains the IgG2F5 epitope ELDKWA and IgG 4E10 epitope NWFDIT–LW, which indicated that the epitope of E10 may overlap with that of 2F5 and/or 4E10. Antibody variable fragment amino acid sequences on these 3 antibodies were compared. Although E10 and 4E10 share the same germline gene family on both VH (IGHV1-69) and VL (IGKV3-20), the amino acid similarities are only 66.14% (VH) and 78.18% (VL), excluding the possibility of E10 as a variant of 4E10 (Figure 4A). To investigate the fine epitope of E10, we did epitope mapping by ELISA with a panel of consensus clade B 15-mer peptides. E10 bound to two linear peptides (P162: SQNQQEKNEQELLEL, P163*Q*EKNEQELLELDKWA) (Figure 4B), suggesting core epitope (QEKNEQELLEL) of E10, which is immediately upstream the 2F5 epitope.

### Mab E10 showed less reactivity with human self-antigen than 2F5

**Figure 5:**
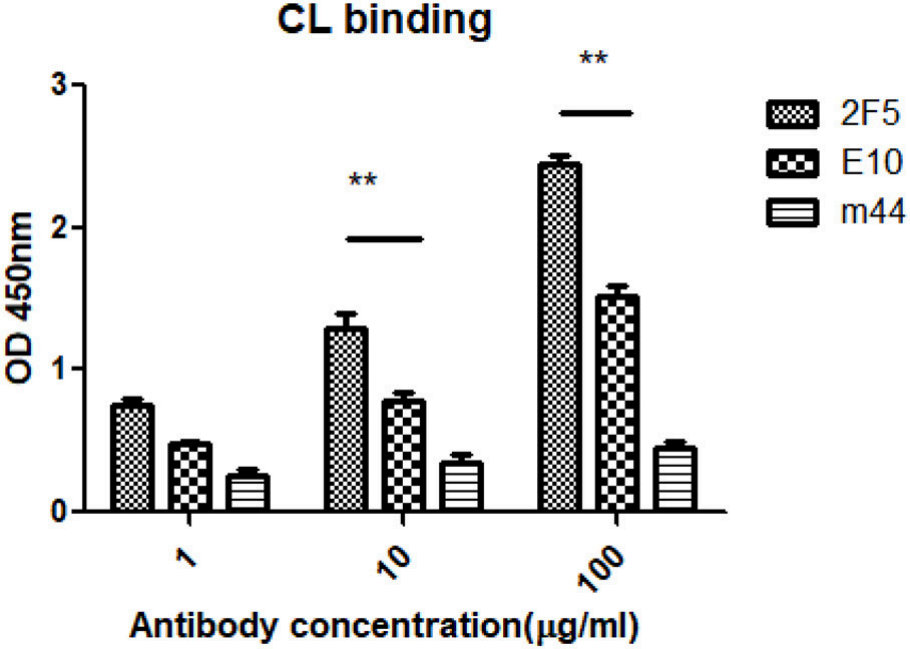
Reactivity of hMAbs with autoantigens. The ELISA for antibody binding to CL was performed as described in section Materials and Methods. Statistical analysis, ^**^P ≤ 0.01.

2F5 has been shown to be reactive with phospholipids, including CL, Ro (SSA), centromere B, and histones, as measured by an AtheNA antinuclear autoantibody assay (ANA) (36). And mAb m44 did not bind to CL (37). We compared in the same experiment binding of m44, 2F5, and E10 in lipid binding assays. E10 binds to CL (Figure 5), whereas Mab E10 showed less reactivity with human self-antigen in comparison with 2F5.

## Materials and methods

### Cell lines, plasmids, peptides, envelope proteins, and antibodies

TZM-bl cell line, TF228 cell line and consensus clade B 15-mer peptides (Cat No. 9480 Lot No. 8 098265) were obtained from the NIH ARRP(AIDS Research and Reference Program). HIV patient PBMC and HIV-1 Env plasmids was kindly provided by Linqi Zhang (Tsinghua University) (38). Antibody expression vectors were kindly provided by Dr. Wilson at University of Chicago. Recombinant plasmids encoding RSC3, deltaRSC3and gp140SF162 trimer were kindly provided by Peter Kwong and John Mascola (Vaccine Research Center, NIAID). Recombinant bal-gp120, gp41-Fc, F7-Fc, RSC3, deltaRSC3 (21), SF162gp140 trimer, IgG b12, and IgG2F5 were expressed in our laboratory using a 293F transient transfection system (Invitrogen) and purified by Immobilized Metal Affinity Chromatography (IMAC) or protein G affinity chromatography.

### HIV envelope specific memory B cell sorting

PBMC was purified by ficoll gradient from HIV infected patient, incubated with 1 μg/mlbiotinylated SF162gp140 trimer at 4°C for 30 min. The PBMC was then washed by PBS and stained with second antibodies:Pacific Blue™ anti-human CD19 (Biolegend, 302224), PE/Cy7 anti-human CD3(Biolegend, 300316), APC/Cy7 anti-human CD14 (Biolegend, 325620), APC anti-human IgG(Biolegend, 409306), 7-AAD(7-Aminoactinomycin D) (Invitrogen, 302232), FITC anti-human CD27(Biolegend, 302806), PerCP/Cy5.5 anti-human IgM (Biolegend, 314512), and R-Phycoerythrin Streptavidin (Jackson immunolab, 016-110-084). SF162gp140-trimerbinding memory B cells (CD19+, IgG+, SF162gp140+7AAD-IgM-CD14-CD3-) were sorted in 96 well PCR plate by FACS AriaIII(BD). The sorted cells were then lysedwith RNA directly reverse transcribed into cDNA according SuperScript™ III CellsDirectcDNA Synthesis System (Invitrogen, 18080-300) manual. Single cell derived cDNA was used for antibody variable gene amplification.

### Antibody gene amplification, vector construction, and protein expression

Antibody variable fragments were amplified using primers and strategies that described by Tiller (30). Paired variable fragments were then cloned into full length IgG eukaryotic transient expression vectors (which were kindly provided by Dr. Wilson at University of Chicago) (31). The vector sequences are available through the NCBI GenBank (accession numbers: FJ475055, FJ475056, and FJ517647). IgG1 were expressed by transfected 293F cells and purified by protein G agarose. Denatured and none-denatured SDS-PAGE was used for antibody purity and size detection.

### Enzyme linked immunosorbent assay

For detection of affinity to envelope proteins, Bal-gp120 and bal-gp41-Fc were coated separately on ELISA plate at 1 μg/ml in phosphate buffer (PH9.6), after PBST wash and skimmed milk blocking, IgGE10 and control antibodieswas added to wells for incubation. Antibodies were three-fold serially diluted starting from 30 μg/ml. Seven dilutions and 3 duplicates were set for each antibody. Anti-human fab-HRP (Jackson ImmunoResearch, 109-036-097) was added as second antibody.EC50 (50% maximum binding) were determined using GraphPad Prism software. For affinity detection against SF162gp140 trimer, RSC3 and deltaRSC3, E10 was diluted staring from 90μg/ml with 8 dilutions. For ELISA based epitope mapping, HIV gp41 peptides (peptide number 121~180, Table S1) were coated separately on ELISA plate at 5 μg/ml in phosphate buffer (PH9.6). Candidate IgGs were diluted to 10 μg/ml for incubation with the coated peptides. Anti-human IgG /Fc-HRP (Jackson ImmunoResearch, 109-006-170) as second antibody. For detection the binding of antibodies against MPER peptide, F7-Fc protein was coated in ELISA plate at 1 μg/ml; biotinylated mAb 2F5, E10, and b12 were then added for incubation with the coated peptides. Antibodies were three-fold serially diluted starting from 50 μg/ml, 3 replicates and 8 dilutions were set for each antibody. Streptavidin-HRP (Jackson ImmunoResearch, 016-030-084) was used as second antibody. F7 (amino acid sequence: QQELLELDKWANLWNWFDISNWLW) is a MPER derived peptide. It was sorted out by mAb E10 against HIV envelop fragment yeast surface display library (39) (manuscript in preparation). The F7 peptide was then fused to the human Fc and expressed in 293F and purified by protein G agarose. For all ELISA experiment in present study, TMB was used as substrate and OD450 were recorded by ELISA reader.

### Flow cytometry assisted detection of E10 binding to F7

MabE10 variable fragments were constructed into SCFV format and co-transformed with linear PYD7 plasmid (39, 40) for *in vivo* recombination. The recombinant clone was screened and induced for E10-SCFV surface expression. Induced E10yeast clone was then incubated with F7-Fc and bal gp120-Fc proteins at 1 μg/ml and then anti human IgG-APC was added as second antibody for staining. Binding were detected by FACS Calibur (BD) and pictures were generated by Flow Jo software.

### Pseudo-virus neutralization assay

A standardized TZM-bl cell line-based Env-pseudotyped neutralization assay was used as previously described (41, 42).

### Antibody-dependent cellular cytotoxicity assay

TF228 was maintained in RPMI1640 containing the same supplements. Heparinized whole blood samples were used to isolate human PBMCs by Ficoll density gradient separation. A flow cytometry-based ADCC assay was set up using TF228 cells as target cells and healthy human PBMCs as effector cells at an T/E ratio of 1/50 (39, 43). In brief, two fluorescent dyes were used to discriminate live and dead cells. PKH-67, a membrane labeling dye, was used to identify the target cells. PKH-67 binds to the cell membrane, and will remain on the membrane even after cell death, avoiding contamination with effector cells. 7-amino-actinomycin-D (7-AAD) is excluded by viable cells, but can stain dead or dying cells by intercalating into double stranded DNA. TF228 cells were stained with PKH-67 for 10 min at RT and 3 ml FBS added to stop the reaction. Pooled PBMCs (3–5 patients) were freshly prepared from five healthy donors and suspended in RPMI 1640 containing 100 U/ml penicillin and 100 μg/ml streptomycin. 2.0 × 10^4^ labeled target cells were dispensed in 50 μl of RPMI 1640 medium in round-bottomed 96-well plate. Fifty microliters of diluted antibodies in triplicate were added to the wells and the plate incubated at 37°C for 15 min in CO_2_ incubator. Fifty microliters of unlabeled pooled PBMCs at a concentration of 2 × 10^7^ cells/ml were added to each well and the plate incubated at 37°C for 4 h in CO_2_ incubator. One micro liter of 7-AAD solution was added to the wells and the plate incubated at 4°C in dark for 15 min. Cell mixtures were analyzed by flow cytometry. Percent ADCC was calculated as [(% experimental lysis – % spontaneous lysis) / (% maximum lysis – % spontaneous lysis)] × 100, in which “% spontaneous lysis” referred to percent lysis of target cells incubated with effector cells in the absence of IgGs, and “% maximum lysis” referred to percent lysis of target cells following heat shock by incubating the cells at 85°C for 30 min.

## Discussion

In present study, we identified a new ADCC antibody that target the MPER region of gp41, adjacent to the epitope of 2F5, 4E10, and 10E8. The study emphasized the MPER region as a dominant ADCC epitope on HIV envelope. HIV MPER region is rich in alpha helix and antibodies (e.g., 2F5, 4E10, and 10E8) that target this region most probably recognize linear epitopes. Unlike the A32-like epitopes which is conformational and hard to mimic, the comparatively simpler structure of MPER may adapt itself in subunit vaccine design. In fact, a number of MPER based subunit vaccine design have already been proved successful at inducing neutralizing and ADCC antibody (15, 44, 45). Further improvement on accessibility and natural conformation simulation of gp41 MPER by rational design may provide effective subunit vaccine that is able to induce potent cross-reactive neutralizing and ADCC antibody *in vivo*.

According to previous reports, most MPER antibodies were limited in potency, and abilities to access MPER on envelope of primary viral isolates. In addition, lipid binding and autoreactivity were also considered to be the characteristics of MPER antibodies. E10 binds to autoantigen, but showed weaker binding activity in comparison with 2F5. Further improvement on accessibility and reduced autoreactivity may help E10 be used in combination with potent neutralizing antibodies in passive treatment. The potent ADCC activity of E10, and the potential roles in combination usage with other potent neutralizing antibodies in passive treatment, emphasizes the contribution of the effector functions of antibodies in prevention against HIV-1 infection.

IgG E10 use a famous V gene segment IGHV1-69 allelic variants. Antibodies derived from this germline V gene tend to have a hydrophobic CDR2 and bind to hydrophobic alpha helix rich portions such as influenza hemagglutinin stem [e.g., monoclonal antibody CR6261 (46), F10 (47), CR9114 (48), FE53 (49), 1009-3B05 (49)] and HIV gp41[e.g., monoclonal antibodyD5 (50), HK20 (51), Fab 8066 (52)] or MPER [e.g., monoclonal antibody4E10 (53, 54)]. IgG E10 from present study is very similar to broad neutralizing antibody 4E10. Both of them use IGHV1-69, are MPER linear epitope specific and be able to mediate ADCC activity except that E10 is less potent in neutralization (though no direct comparison).The reason why a little shift on epitope results in different neutralization activity is worthy investigation. In present study, the characterization of IgG E10 is not complete to some extent; we only tested neutralization of E10 on 6 HIV pseudo virus isolates due to reagent and platform availability, it is necessary to investigate the neutralization breath and potency by a larger panel of HIV isolates.

## Author contributions

ZY, XL, MZ, ZS, and WY conceived and designed the experiment. XL, ZY, WT, ZS, and JL performed the experiments. ZY, MZ, WY, and XL wrote the paper.

### Conflict of interest statement

The authors declare that the research was conducted in the absence of any commercial or financial relationships that could be construed as a potential conflict of interest.

## Abbreviations

ADCC: Antibody-Dependent Cellular Cytotoxicity
CDR: Cluster Differentiation Region
ELISA: Enzyme Linked Immunosorbent Assay
FACS: Flow-cytometry Assisted Cell Sorting
mAbs: Monoclonal Antibodies
MPER: Membrane Proximal External Region
PBMC: Peripheral Blood Mononuclear Cell
SCFV: Single Chain Fragment of Variable.
