# Supplementary material for: Identification of a HIV Gp41-Specific Human Monoclonal Antibody With Potent Antibody-Dependent Cellular Cytotoxicity
Source: Front Immunol. 2018 Nov 16;9:2613. doi: 10.3389/fimmu.2018.02613 (PMC6251304; doi:10.3389/fimmu.2018.02613)
Supplement: Supplementary file 1 [file Table_1.DOC]

**Supplementary materials:**

TableS1. HIV-1 Consensus B Env Peptides (15 mer)(peptide 121~180)

| **Cat No. 9480 Lot No. 8 098265** | | | | | | |  | | | |
| --- | --- | --- | --- | --- | --- | --- | --- | --- | --- | --- |
| **Number** | **Cat No.** | **PEPTIDE NAME** | **SEQUENCE** | **Mol.Wt (g/mol)** | **HPLC Purity %** | **Peptide Content** | **Water** | **PBS** | **10% Acetic Acid** | **DMSO** |
| 121 | 8883 | HIV-1 Con Subtype B Env (15-mer) | KYKVVKIEPLGVAPT | 1640.95 | 86.1% | 78.3% | + | + | + | + |
| 122 | 8884 | HIV-1 Con Subtype B Env (15-mer) | VKIEPLGVAPTKAKR | 1605.96 | 93.2% | 73.8% | + | + | + | + |
| 123 | 8885 | HIV-1 Con Subtype B Env (15-mer) | PLGVAPTKAKRRVVQ | 1618.98 | 85.5% | 74.0% | + | + | + | + |
| 124 | 8886 | HIV-1 Con Subtype B Env (15-mer) | APTKAKRRVVQREKR | 1822.09 | 92.4% | 66.7% | + | + | + | + |
| 125 | 8887 | HIV-1 Con Subtype B Env (15-mer) | AKRRVVQREKRAVGI | 1765.07 | 89.0% | 68.9% | + | + | + | + |
| 126 | 8888 | HIV-1 Con Subtype B Env (15-mer) | VVQREKRAVGIGAMF | 1659.91 | 96.8% | 78.5% | + | + | + | + |
| 127 | 8889 | HIV-1 Con Subtype B Env (15-mer) | EKRAVGIGAMFLGFL | 1607.86 | 81.9% | 82.5% | - | - | + | + |
| 128 | 8890 | HIV-1 Con Subtype B Env (15-mer) | VGIGAMFLGFLGAAG | 1379.71 | 91.6% | 85.8% | - | - | - | + |
| 129 | 8891 | HIV-1 Con Subtype B Env (15-mer) | AMFLGFLGAAGSTMG | 1429.66 | 92.8% | 86.3% | - | - | - | + |
| 130 | 8892 | HIV-1 Con Subtype B Env (15-mer) | GFLGAAGSTMGAASM | 1327.58 | 95.0% | 85.4% | + | + | + | + |
| 131 | 8893 | HIV-1 Con Subtype B Env (15-mer) | AAGSTMGAASMTLTV | 1367.64 | 98.6% | 85.7% | + | - | + | + |
| 132 | 8894 | HIV-1 Con Subtype B Env (15-mer) | TMGAASMTLTVQARQ | 1564.77 | 84.1% | 87.3% | + | + | + | + |
| 133 | 8895 | HIV-1 Con Subtype B Env (15-mer) | ASMTLTVQARQLLSG | 1574.83 | 96.7% | 87.4% | + | + | + | + |
| 134 | 8896 | HIV-1 Con Subtype B Env (15-mer) | LTVQARQLLSGIVQQ | 1652.94 | 89.1% | 87.9% | + | + | + | + |
| 135 | 8897 | HIV-1 Con Subtype B Env (15-mer) | ARQLLSGIVQQQNNL | 1680.9 | 88.5% | 88.1% | + | - | + | + |
| 136 | 8898 | HIV-1 Con Subtype B Env (15-mer) | LSGIVQQQNNLLRAI | 1665.92 | 86.4% | 88.0% | - | - | - | + |
| 137 | 8899 | HIV-1 Con Subtype B Env (15-mer) | VQQQNNLLRAIEAQQ | 1751.91 | 96.5% | 88.5% | + | + | + | + |
| 138 | 8900 | HIV-1 Con Subtype B Env (15-mer) | NNLLRAIEAQQHLLQ | 1759.94 | 96.2% | 83.7% | + | + | + | + |
| 139 | 8901 | HIV-1 Con Subtype B Env (15-mer) | RAIEAQQHLLQLTVW | 1804.98 | 88.8% | 84.1% | + | + | + | + |
| 140 | 8902 | HIV-1 Con Subtype B Env (15-mer) | AQQHLLQLTVWGIKQ | 1761.97 | 82.5% | 83.8% | - | - | - | + |
| 141 | 8903 | HIV-1 Con Subtype B Env (15-mer) | LLQLTVWGIKQLQAR | 1766.03 | 91.3% | 83.8% | - | - | - | + |
| 142 | 8904 | HIV-1 Con Subtype B Env (15-mer) | TVWGIKQLQARVLAV | 1680.99 | 93.7% | 83.1% | - | - | - | + |
| 143 | 8905 | HIV-1 Con Subtype B Env (15-mer) | IKQLQARVLAVERYL | 1799.05 | 84.7% | 79.8% | + | - | + | + |
| 144 | 8906 | HIV-1 Con Subtype B Env (15-mer) | QARVLAVERYLKDQQ | 1815.98 | 91.0% | 79.9% | + | + | + | + |
| 145 | 8907 | HIV-1 Con Subtype B Env (15-mer) | LAVERYLKDQQLLGI | 1757.97 | 83.7% | 83.7% | - | + | + | + |
| 146 | 8908 | HIV-1 Con Subtype B Env (15-mer) | RYLKDQQLLGIWGCS | 1778.88 | 96.1% | 83.9% | + | + | + | + |
| 147 | 8909 | HIV-1 Con Subtype B Env (15-mer) | DQQLLGIWGCSGKLI | 1629.82 | 85.8% | 87.7% | + | + | + | + |
| 148 | 8910 | HIV-1 Con Subtype B Env (15-mer) | LGIWGCSGKLICTTT | 1551.75 | 98.9% | 87.2% | + | + | + | + |
| 149 | 8911 | HIV-1 Con Subtype B Env (15-mer) | GCSGKLICTTTVPWN | 1578.73 | 82.9% | 87.4% | + | + | + | + |
| 150 | 8912 | HIV-1 Con Subtype B Env (15-mer) | KLICTTTVPWNASWS | 1705.83 | 82.4% | 88.2% | + | + | + | + |
| 151 | 8913 | HIV-1 Con Subtype B Env (15-mer) | TTTVPWNASWSNKSL | 1690.81 | 98.7% | 88.1% | + | + | + | + |
| 152 | 8914 | HIV-1 Con Subtype B Env (15-mer) | PWNASWSNKSLDEIW | 1831.82 | 87.0% | 88.9% | - | - | - | + |
| 153 | 8915 | HIV-1 Con Subtype B Env (15-mer) | SWSNKSLDEIWDNMT | 1824.77 | 80.1% | 88.9% | - | - | + | + |
| 154 | 8916 | HIV-1 Con Subtype B Env (15-mer) | KSLDEIWDNMTWMEW | 1982.83 | 81.5% | 89.7% | - | - | - | + |

| **Cat No. 9480 Lot No. 8 098265** | | | | | | |  | | | |
| --- | --- | --- | --- | --- | --- | --- | --- | --- | --- | --- |
| **Number** | **Cat No.** | **PEPTIDE NAME** | **SEQUENCE** | **Mol.Wt (g/mol)** | **HPLC Purity %** | **Peptide Content** | **Water** | **PBS** | **10% Acetic Acid** | **DMSO** |
| 155 | 8917 | HIV-1 Con Subtype B Env (15-mer) | EIWDNMTWMEWEREI | 2066.86 | 87.4% | 90.1% | - | - | - | + |
| 156 | 8918 | HIV-1 Con Subtype B Env (15-mer) | NMTWMEWEREIDNYT | 2016.81 | 85.3% | 89.8% | - | + | + | + |
| 157 | 8919 | HIV-1 Con Subtype B Env (15-mer) | MEWEREIDNYTSLIY | 1960.85 | 88.2% | 89.6% | - | - | + | + |
| 158 | 8920 | HIV-1 Con Subtype B Env (15-mer) | REIDNYTSLIYTLIE | 1841.9 | 85.7% | 89.0% | - | - | - | + |
| 159 | 8921 | HIV-1 Con Subtype B Env (15-mer) | NYTSLIYTLIEESQN | 1786.82 | 87.9% | 88.7% | - | - | - | + |
| 160 | 8922 | HIV-1 Con Subtype B Env (15-mer) | LIYTLIEESQNQQEK | 1834.89 | 80.2% | 89.0% | + | + | + | + |
| 161 | 8923 | HIV-1 Con Subtype B Env (15-mer) | LIEESQNQQEKNEQE | 1844.8 | 80.1% | 89.0% | + | - | - | + |
| 162 | 8924 | HIV-1 Con Subtype B Env (15-mer) | SQNQQEKNEQELLEL | 1828.84 | 100.0% | 88.9% | + | + | + | + |
| 163 | 8925 | HIV-1 Con Subtype B Env (15-mer) | QEKNEQELLELDKWA | 1871.89 | 90.7% | 84.6% | - | - | + | + |
| 164 | 8926 | HIV-1 Con Subtype B Env (15-mer) | EQELLELDKWASLWN | 1872.89 | 86.3% | 89.2% | - | - | - | + |
| 165 | 8927 | HIV-1 Con Subtype B Env (15-mer) | LELDKWASLWNWFDI | 1934.93 | 88.9% | 89.5% | - | - | - | + |
| 166 | 8928 | HIV-1 Con Subtype B Env (15-mer) | KWASLWNWFDITNWL | 1978.95 | 100.0% | 89.7% | + | - | + | + |
| 167 | 8929 | HIV-1 Con Subtype B Env (15-mer) | LWNWFDITNWLWYIK | 2097.02 | 82.3% | 90.2% | - | - | - | + |
| 168 | 8930 | HIV-1 Con Subtype B Env (15-mer) | FDITNWLWYIKIFIM | 2002.01 | 80.9% | 89.8% | - | + | + | + |
| 169 | 8931 | HIV-1 Con Subtype B Env (15-mer) | NWLWYIKIFIMIVGG | 1851.97 | N/A | 89.0% | - | - | - | + |
| 170 | 8932 | HIV-1 Con Subtype B Env (15-mer) | YIKIFIMIVGGLIGL | 1648.95 | N/A | 87.9% | - | - | - | + |
| 171 | 8933 | HIV-1 Con Subtype B Env (15-mer) | FIMIVGGLIGLRIVF | 1646.96 | N/A | 87.8% | - | - | - | + |
| 172 | 8934 | HIV-1 Con Subtype B Env (15-mer) | VGGLIGLRIVFAVLS | 1512.91 | 99.2% | 86.9% | - | - | - | + |
| 173 | 8935 | HIV-1 Con Subtype B Env (15-mer) | IGLRIVFAVLSIVNR | 1669.01 | 87.7% | 83.0% | - | - | - | + |
| 174 | 8936 | HIV-1 Con Subtype B Env (15-mer) | IVFAVLSIVNRVRQG | 1669.98 | 94.6% | 83.0% | - | - | - | + |
| 175 | 8937 | HIV-1 Con Subtype B Env (15-mer) | VLSIVNRVRQGYSPL | 1699.94 | 84.2% | 83.3% | - | - | + | + |
| 176 | 8938 | HIV-1 Con Subtype B Env (15-mer) | VNRVRQGYSPLSFQT | 1750.89 | 82.0% | 83.7% | + | + | + | + |
| 177 | 8939 | HIV-1 Con Subtype B Env (15-mer) | RQGYSPLSFQTRLPA | 1719.88 | 83.8% | 83.4% | + | + | + | + |
| 178 | 8940 | HIV-1 Con Subtype B Env (15-mer) | SPLSFQTRLPAPRGP | 1622.86 | 84.7% | 82.6% | + | + | + | + |
| 179 | 8941 | HIV-1 Con Subtype B Env (15-mer) | FQTRLPAPRGPDRPE | 1735.89 | 90.7% | 79.2% | + | + | + | + |
| 180 | 8942 | HIV-1 Con Subtype B Env (15-mer) | LPAPRGPDRPEGIEE | 1631.79 | 90.2% | 82.7% | + | + | + | + |

1 Determination of solubility: appr. 0.25 mg of the respective peptide was incubated with 1ml of solvent. Solubility was assessed by visual inspection of the resulting solution/ suspension. ( "+": complete dissolution, "-": incomplete dissolution)

NOTE: Peptides that are difficult to solubilize can almost always be dissolved in DMSO. Once a peptide is in solution, the DMSO can be slowly diluted with aqueous medium. Care must be taken to ensure that the peptide does not begin to precipitate out of solution.
